# The association of sleep-disordered breathing with high cerebral pulsatility might not be related to diffuse small vessel disease. A pilot study

**DOI:** 10.1186/s13104-015-1481-5

**Published:** 2015-09-29

**Authors:** Pablo R. Castillo, Oscar H. Del Brutto, María de la Luz Andrade, Mauricio Zambrano, Juan A. Nader

**Affiliations:** Division of Sleep Medicine, Mayo Clinic, 4500 San Pablo Rd, Jacksonville, FL 32224 USA; School of Medicine, Universidad Espíritu Santo-Ecuador, Guayaquil, Ecuador; Department of Neurology, Hospital Médica Sur, Mexico City, Mexico; Community Center, The Atahualpa Project, Atahualpa, Ecuador

**Keywords:** Cerebral pulsatility, Cerebral small vessel disease, Pulsatility index, Sleep-disordered breathing, Transcranial Doppler, White matter hyperintensities

## Abstract

**Background:**

In a population-based sampling study conducted in community-dwelling older adults living in rural Ecuador, we aimed to assess the relation among sleep-disordered breathing, cerebral pulsatility index, and diffuse small vessel disease.

**Methods:**

Of 25 participants, 9 (36 %) had moderate-to-severe sleep-disordered breathing, characterized by an apnea/hypopnea index ≥15 per hour, and 10 (40 %) had moderate-to-severe white matter hyperintensities, graded according to the modified Fazekas scale. Mean (SD) pulsatility index in the middle cerebral artery was 1.18 (0.19) and positively correlated with the apnea/hypopnea index (*R* = .445, *P* = .03, [Pearson’s correlation coefficient]). The middle cerebral artery pulsatility index was increased in persons with moderate-to-severe sleep-disordered breathing compared with persons who had none-to-mild sleep-disordered breathing (mean [SD] 1.11 [0.12] vs. 1.3 [0.23], *P* = .01). No significant differences were found in the prevalence of moderate-to-severe white matter hyperintensities across groups of sleep-disordered breathing (*P* = .40) or in the mean apnea/hypopnea index across groups of persons with none-to-mild or moderate-to-severe white matter hyperintensities (*P* = .16).

**Conclusions:**

This pilot study shows that moderate-to-severe sleep-disordered breathing correlates with cerebral pulsatility, but such association might be independent of diffuse small vessel disease.

## Background

The association of sleep-disordered breathing (SDB) with cerebral pulsatility has been reported to be linked to small vessel disease (SVD) [1]. However, a high cerebral pulsatility index (PI) may also result from large-artery stiffness or other hemodynamic factors and is not specific for SVD [2, 3]. Therefore, a high PI should not be considered a proxy of SVD in persons with SBD until more evidence is available. The complex—and probably bidirectional—relation between SDB and stroke is linked not only to SVD, but to other pathogenetic mechanisms [4, 5], which could continue to be unrecognized if a complete investigation is not performed on the basis of this assumption.

The Atahualpa Project is an ongoing population-based study designed to reduce the increasing burden of non-communicable diseases—including sleep disorders—in rural Ecuador [6]. Preliminary findings from our cohort suggest an association between non-breathing-related sleep symptoms with cardiovascular risk factors [7], overt stroke [8] and silent imaging markers of SVD [9]. Here, we aimed to assess the relationship between SDB, cerebral pulsatility, and diffuse SVD in a random sample of individuals enrolled in the Atahualpa Project.

## Methods

The institutional review board of Hospital-Clínica Kennedy in Guayaquil, Ecuador (Federalwide Assurance 00006867) approved the study protocol and the written informed consent. Fifty randomly selected individuals were invited to undergo TCD. They were selected by the use of the Random Integer Generator (https://www.random.org/integers/) from a total of 239 Atahualpa residents identified during a door-to-door survey and fulfilling the following criteria: (1) age ≥60 years, (2) clinical stroke-free status, (3) brain MRI performed in the previous 6 months, (4) absence of significant (>50 %) stenosis of all major intracranial vessels on magnetic resonance angiography, and (5) no documented atrial fibrillation.

**Fig. 1:**
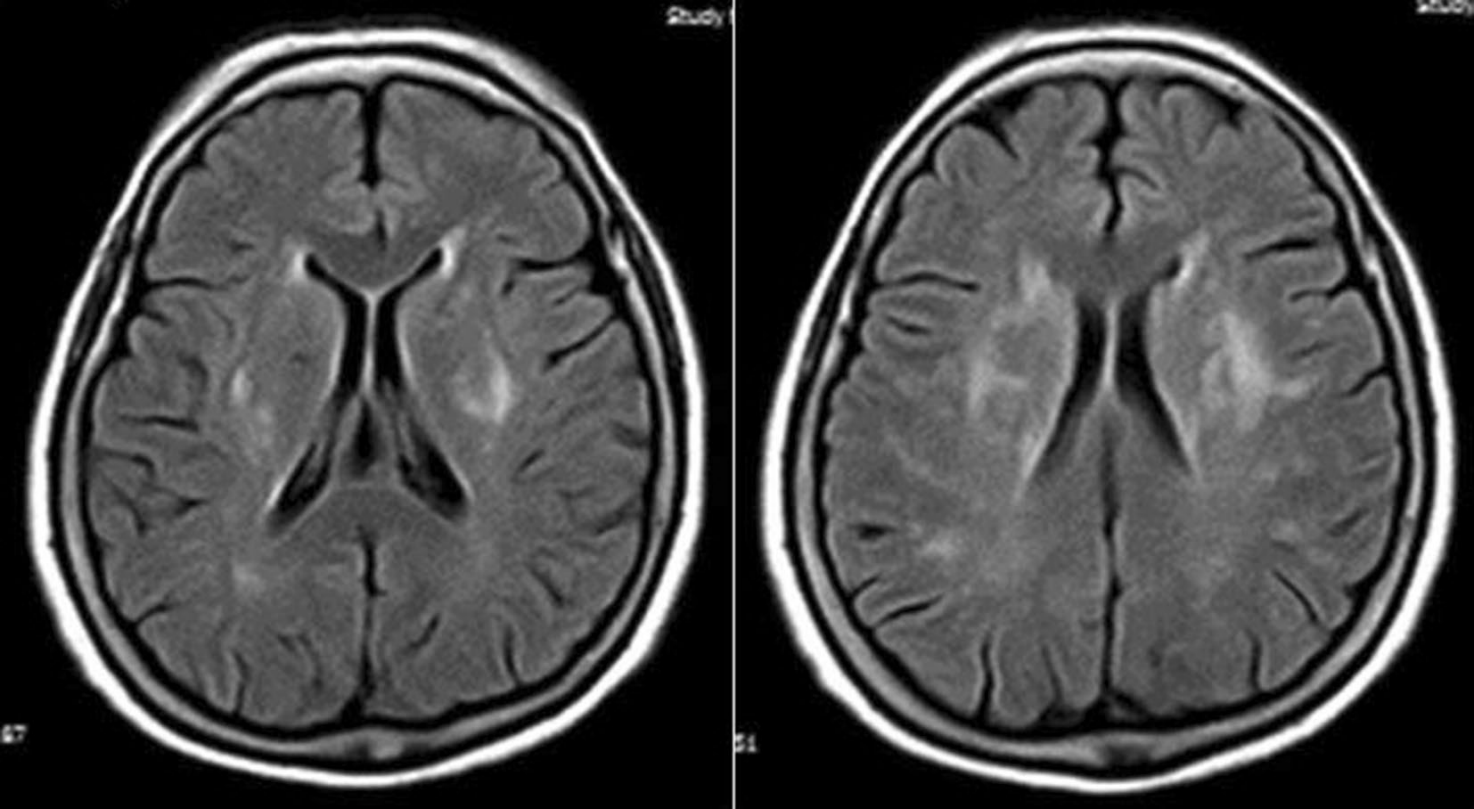
Fluid-attenuated inversion recovery magnetic resonance imaging (TR/TE/TI = 9000/120/2500 ms). Both images show severe white matter hyperintensities of presumed vascular origin according to the modified Fazekas scale. Of note is the extension of periventricular lesions into the subcortical white matter and large confluent subcortical foci

As previously detailed, the neuroimaging studies were performed with use of a Philips Intera 1.5T MRI machine (Philips Healthcare) at the Hospital-Clínica Kennedy, Guayaquil [10]. For the current study, the primary focus was on white matter hyperintensities (WMHs) of presumed vascular origin, defined as lesions appearing hyperintense on T2-weighted images that remained bright on fluid-attenuated inversion recovery (without cavitation) and graded into mild, moderate, and severe according to the modified Fazekas scale [11]. Mild cases present with periventricular caps or thin lesions and punctate hyperintensities in subcortical white matter. In moderate cases, there is a smooth periventricular halo, and subcortical foci begin to merge. Severe cases are characterized by extension of periventricular lesions into the subcortical white matter and large confluent subcortical foci (Fig. 1).

Diagnostic single-night PSG was performed at the sleep unit of the Atahualpa Project Community Center with use of an Embletta X100 Comprehensive Portable PSG System (Embla Systems, Inc). Measured parameters included electroencephalography, electro-oculography, electrocardiography, chin and leg myography, nasal airflow pressure, abdominal and thoracic inductive plethysmography, and pulse oximetry. PSG results were scored by certified polysomnographic technicians following the rules contained in *The American Academy of Sleep Medicine (AASM) Manual for the Scoring of Sleep and Associated Events* (version 2.0) [12]. Data were reviewed by a board-certified sleep medicine neurologist (P.R.C.) to whom all other information was masked.

**Fig. 2:**
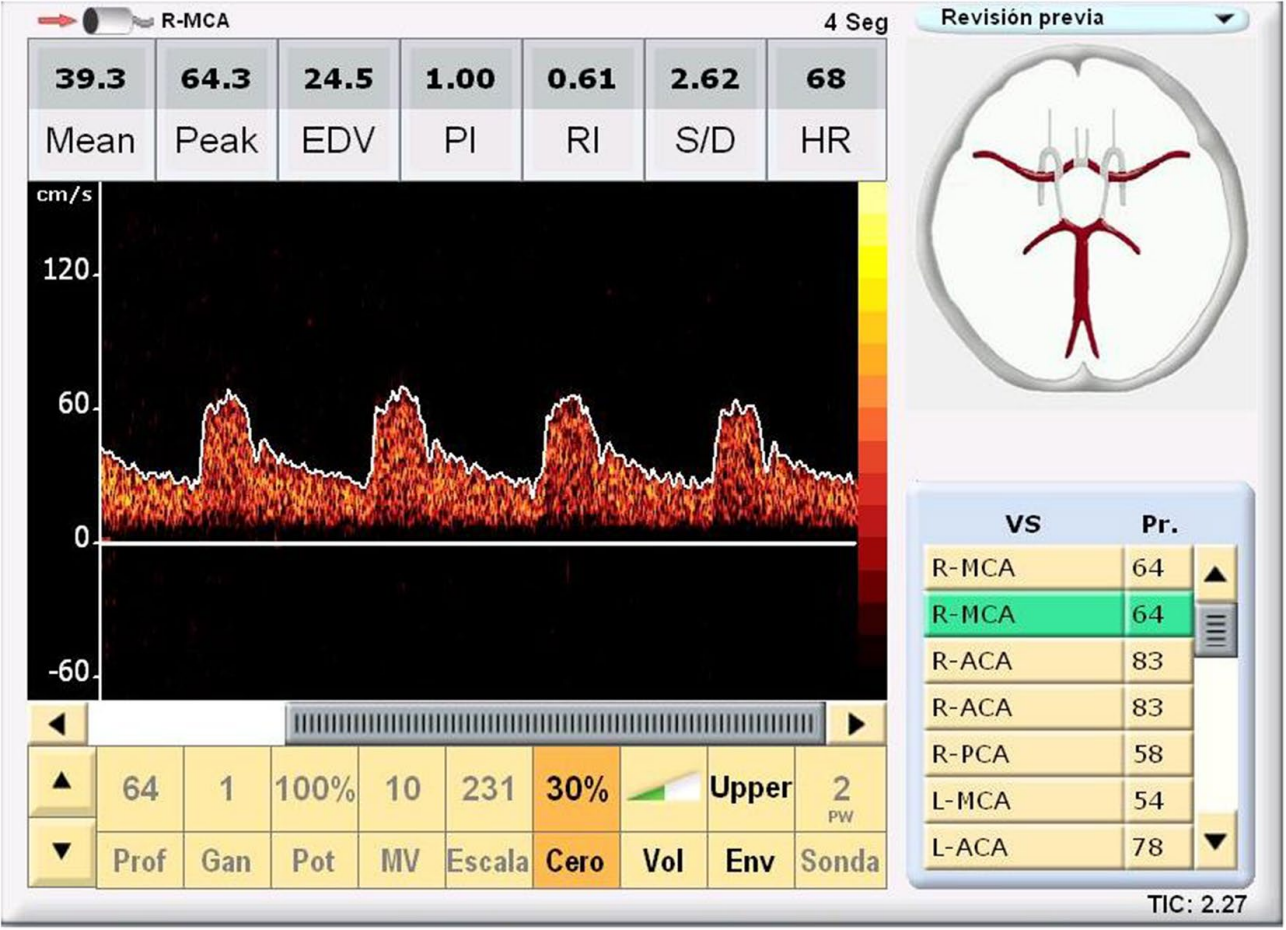
Screen of the Transcranial Doppler Study Performed in a Study Participant. Image showing the parameters evaluated for each artery (in this particular image, the right middle cerebral artery)

With the use of a SONARA portable system (VIASYS Healthcare, Inc) and a 2-MHz probe, a certified sonographer (J.A.N.) performed all TCD examinations following a well-known power motion mode Doppler/spectral TCD protocol [13]. For the present study, peak systolic velocity, end-diastolic velocity, mean flow velocity, and PI of both middle cerebral arteries (MCAs) were analyzed (Fig. 2). The latter was calculated with the Gosling equation (peak systolic velocity—end-diastolic velocity/mean flow velocity). Mean values for each of these variables were calculated through averaging both MCAs unless flows from only one artery could be evaluated because of poor insonation through the contralateral transtemporal window.

All analyses were carried out by using STATA version 13 (Stata Corp LC). Descriptive statistics are presented as mean (SD) for continuous variables and as percentage (95 % CI) for categorical variables. Continuous variables of the PI and the AHI were compared by the use of the Pearson’s correlation coefficient. A *P* value less than .05 was considered significant. Regression models were not constructed.

## Results

Of the 50 invited persons, 40 agreed to participate and 38 had adequate PSG recordings to allow for proper data assessment. Thirteen of the 38 persons had absent transtemporal windows precluding insonation of both MCAs and therefore were excluded. (These high rates of absent windows were related to increased thickness and heterogeneity of temporal squama in our population of Amerindians [14, 15].

The final 25 participants had a mean (SD) age of 73.1 (7.2) years, and 13 (52 %) were women. Only one participant was a smoker, four had a body mass index ≥30 kg/m^2^, 16 had arterial hypertension, and three had diabetes mellitus. Nine persons (36 %) had moderate-to-severe SDB [apnea/hypopnea index (AHI) ≥ 15/h] and 10 (40 %) had moderate-to-severe WMHs (six of these individuals had blood pressure ≥140/90 mm Hg). Mean (SD) MCA PI in the overall sample was 1.18 (0.19) and positively correlated with the AHI [*R* = .445, *P* = .03 (Pearson’s correlation coefficient)].

Table 1 summarizes characteristics of the 25 study participants. No differences were found in demographic characteristics or cardiovascular risk factors across the two groups of AHI severity. PSGs showed greater numbers of total sleep arousals per hour and lower oxygen saturation among persons with moderate-to-severe SDB. The PI was the only TCD variable that was significantly increased in persons with moderate-to-severe SDB compared with those who had none-to-mild SDB (*P* = .01). Ten (40 %) of the 25 participants had moderate-to-severe WMHs, which were more prevalent among persons with moderate-to-severe SDB; however, this difference did not reach significance. In addition, stratification according to WMH severity showed no significant differences in the mean (SD) AHI across groups of persons with none-to-mild or moderate-to-severe WMHs (13.7 [13] vs. 22.6 [17.8], *P* = .16).

**Table 1:** Characteristics of study participants according to the apnea-hypopnea index

	Total series (n = 25)	AHI < 15 (n = 16)	AHI ≥ 15 (n = 9)	*P* value
Age, mean (SD), years	73.1 (7.2)	72 (5.8)	75.1 (8.7)	.295
Women, no. (%)	13 (52)	8 (50)	5 (56)	1.0
BMI, mean (SD), kg/m^2^	26.1 (5)	25.8 (4.4)	26.7 (6)	.671
Arterial hypertension, no. (%)	16 (64)	11 (69)	5 (56)	.671
Diabetes mellitus, no. (%)	3 (12)	2 (13)	1 (11)	1.0
Sleep efficiency, mean (SD)	69.6 (17.8)	66.6 (18.7)	74.9 (14.8)	.265
Total arousals per h, mean (SD)	19.7 (13.3)	12 (5.1)	33.3 (12.4)	.0001
Oxygen saturation, mean (SD)	95.1 (1.9)	96 (1.4)	93.5 (1.5)	.0001
MCA peak systolic velocity, mean (SD)	76 (21.3)	77.3 (21.8)	73.6 (20.3)	.680
MCA end-diastolic velocity, mean (SD)	25.5 (9.3)	27.1 (9)	22.6 (9.2)	.246
MCA mean flow velocity, mean (SD)	43.8 (13.4)	45.5 (13.5)	40.7 (12.8)	.394
MCA pulsatility index, mean (SD)	1.18 (0.19)	1.11 (0.12)	1.30 (0.23)	.012
Moderate-to-severe WMH, no. (%)	10 (40)	5 (31)	5 (56)	.397

Continuous variables were compared by linear models (analysis of variance) and categorical variables by *x* ^2^ or Fisher exact test as appropriate

*AHI* apnea/hypopnea index, *BMI* body mass index, *MCA* middle cerebral artery, *WMH* white matter hyperintensities

## Discussion

The present pilot study shows that moderate-to-severe SDB correlates with cerebral pulsatility, as demonstrated by a direct relation between AHI and MCA PI values. However, such association might be independent of WMH severity and thus may not be mediated by the presence of diffuse cerebral SVD. SDB has an independent effect on arterial stiffness, which could contribute to the mechanisms accounting for SDB-associated strokes [16]. Yet, recent Korean studies independently showed a significant association of SDB with lacunar infarcts [17] and WMH [18]; unfortunately, their studies did not include TCD examinations and the relation with PI could not be assessed.

A limitation of our study is the small sample size, precluding the construction of regression models adjusted for potentially confounding variables. However, the population-based random sampling and case–control design, together with both use of validated protocols for PSG and TCD interpretation and the quality of MRI scans, are indicative for the strengths of our findings. Further large-scale, longitudinal studies in our population will help to clarify whether the association between SDB and cerebral pulsatility is related to the severity of diffuse SVD or if the apparent association actually occurs because of different pathogenetic mechanisms.

## Abbreviations

AHI: apnea/hypopnea index
MCA: middle cerebral artery
MRI: magnetic resonance imaging
PI: pulsatility index
PSG: polysomnography
SDB: sleep-disordered breathing
SVD: small vessel disease
TCD: transcranial Doppler
WMH: white matter hyperintensities
